# The importance of functional tests to assess the effect of a new *CFTR* variant when genotype–phenotype correlation is not possible

**DOI:** 10.1002/ccr3.760

**Published:** 2017-03-30

**Authors:** Alexandre Hinzpeter, Marie‐Pierre Reboul, Isabelle Callebaut, Cécile Zordan, Bruno Costes, Julie Guichoux, Albert Iron, Didier Lacombe, Natacha Martin, Benoit Arveiler, Pascale Fanen, Patricia Fergelot, Emmanuelle Girodon

**Affiliations:** ^1^ Institut Necker Enfants MaladesInserm U1151 and Université Paris DescartesParisFrance; ^2^ CHU de BordeauxService de Génétique MédicaleBordeauxFrance; ^3^ Département de Biologie StructuraleCNRS UMR7590 and Universités Paris 6 et Paris 7ParisFrance; ^4^ CHU de BordeauxService de Pédiatrie MédicaleBordeauxFrance; ^5^ Inserm U955 and Université Paris‐EstCréteilFrance; ^6^ Inserm, U1211 and Université de BordeauxBordeauxFrance; ^7^ Département de GénétiqueHôpital Henri Mondor, AP‐HPCréteilFrance; ^8^ Laboratoire de Génétique et Biologie MoléculairesHôpital Cochin, AP‐HPParisFrance

**Keywords:** CFTR, cystic fibrosis, functional studies, genotype–phenotype correlation, p.Cys1355Phe

## Abstract

In vitro functional tests aimed to investigate CFTR dysfunction appear critical to help elucidate the functional impact of new variants of uncertain clinical significance and solve inconclusive cases, especially in early deceased newborns.

## Introduction

More than 2000 sequence variants in the cystic fibrosis transmembrane conductance regulator gene (*CFTR*; NM_000492.3; MIM# 602421) have been reported in patients with cystic fibrosis (CF; MIM# 219700) 1, one of the most frequent lifeshortening autosomal recessive diseases in the Caucasian population, and in a broad spectrum of phenotypes, so called CFTR‐related disorders 2 (Cystic Fibrosis Mutation Database (CFMDB)). Phenotype variability is mostly explained by *CFTR* mutational and genotype heterogeneity, interplaying with other genetic and environmental factors. Establishing a CF diagnosis is critical for patients and their families, in order to provide appropriate medical care, follow‐up, and genetic counseling. Laboratory criteria include a positive sweat test (chloride value above 60 mmol/L) and/or abnormal values of electrophysiological measurements and/or the presence of two CF‐causing variants, one in each *CFTR* gene copy 3. However, the diagnosis may be inconclusive in a number of situations, especially in the context of newborn screening 4, when electrophysiological tests are equivocal or not possible, or when the pathogenicity of variants is uncertain. In vitro functional tests may help elucidate the functional impact of variants on *CFTR* mRNA splicing and protein localization, processing and function, and hereby solve inconclusive cases. So far, only a minority of variants has been characterized by functional studies 5.

We report on a premature newborn who was highly suspected of having CF and was found to carry two *CFTR* variants, one of which being a new missense variant of uncertain clinical significance. Because of an early death, in vitro functional studies were performed to investigate the pathogenicity and molecular mechanism of this defect.

## Material and Methods

### Case report

The patient was a premature newborn who presented with high immunoreactive trypsin at CF newborn screening but had not had any sweat test because of prematurity and early death at D13. Newborn screening for 30 frequent variants (Elucigene CF30v2, Manchester, UK) identified the heterozygous CF‐causing variant c.2051_2052delinsG, p.Lys684Serfs (2183AA>G).

The infant was born by cesarean section at 24 weeks of gestation due to premature rupture of membranes and failure of tocolysis in her mother. The newborn was admitted in the intensive care unit after birth. She was eutrophic with hyaline membrane disease and received a tri‐antibiotic therapy because of severe infection with major leucocytosis. At 48 h of life, respiratory function deteriorated with pulmonary arterial hypertension that required high‐frequency ventilation and inhaled nitric oxide (NO) concomitant with hemodynamic instability, which was controlled by dopamine. In addition, the detection of *Candida albicans* in tracheal secretions led to administration of triflucan. The clinical condition gradually improved, conventional ventilation resumed, and the inhaled NO and dopamine were discontinued. However, on D12, respiratory and hemodynamic status deteriorated sharply and abdominal ultrasonography revealed the presence of necrotizing enterocolitis. The baby was moved back to high‐frequency ventilation, inhaled NO, and dopamine. Her hemodynamic instability could no longer be controlled, and she died on D13 as a result of multiple organ failure.

Regarding the family history, the father, who is Italian and has three healthy children from a previous marriage (aged 25, 22, and 17 years), had recurrent bronchitis, nasal obstruction, acute pancreatitis, and gastrointestinal malabsorption. The mother, who has two healthy daughters from a previous marriage (aged 20 and 9 years), had pyelonephritis and a spontaneous miscarriage 3 years ago.

### CFTR gene analysis and *in silico* studies

Genomic DNA sample was extracted from blood spots collected for newborn screening. Sanger sequencing of the *CFTR* gene promoter, the 27 coding exons, and intron/exon boundaries (NG_016465.1) was performed using an Applied Biosystems‐3130 DNA Analyzer (Foster City, CA). Large rearrangements were searched for using the SALSA MLPA kit (P091‐C1 CFTR, MRC Holland, Amsterdam, the Netherlands). Exon numbers and sequence variants were named according to HGVS recommendations, whereas traditional mutations names were given in brackets.

*In silico* analysis was performed using Alamut^®^ Software v2.7 (Interactive Biosoftware, Rouen, France).

To gain insight into the consequences of the mutant protein, we also considered its position within a model of the CFTR membrane‐spanning domain and nucleotide‐binding domain 2 (MSD:NBD) 3D structure 6.

### Cell culture and transfection

BEAS‐2B cells were grown in LHC8 media complemented with 10% SVF, whereas HEK293 and HeLa cells were grown in DMEM media complemented with 10% SVF, all from Gibco^™^ (Courtaboeuf, France). Cells were maintained at 37°C, 5% CO_2_. Subconfluent cell cultures were transfected with Turbofect following manufacturer's instructions (ThermoFisher Scientific, Courtaboeuf, France).

### Minigene construct

*CFTR* exon 25 and flanking intronic sequences were PCR amplified using the following forward and reverse primers: ccgctcgagTgcattcagttgtgttggaa and gcgctctagaCatgcttatggtataaatgggatac containing restriction sites for, respectively, *XhoI* and *XbaI*. PCR products were gel purified and digested using both *XhoI* and *XbaI* before being ligated in pET01 minigene vector (MoBiTec‐GmbH, Göttingen, Germany). Obtained clones were fully sequenced to assess the absence of any other sequence variant.

### Mutagenesis

Variant c.4064G>T, p.Cys1355Phe, was generated using the following primer couple: CCACAAGCAGTTGATGTTCTTGGCTAGATCTGTTC and GAACAGATCTAGCCAAGAACATCAACTGCTTGTGG. Mutagenesis was performed on CFTR‐wild‐type (WT) cDNA cloned in pTracer vector and CFTR exon 25 minigene cloned in pET01 vector using the QuickChange XL mutagenesis kit (Agilent, Les Ulis, France). Obtained clones were fully sequenced.

### Hybrid minigene splicing assay

BEAS‐2B cells were plated on six‐well plates and transfected with WT or mutant minigenes. RT‐PCR analysis was performed as previously described 7.

### Immunoprecipitation and Western Blot analysis

HeLa cells were plated on 100‐mm dishes and transfected with CFTR‐WT or mutant CFTR. Immunoprecipitation and Western blot were performed as previously described 7.

### Immunocytochemistry

HeLa cells were transfected on glass coverslips with CFTR‐WT or CFTR‐p.Cys1355Phe and fixed using methanol. Immunocytochemistry was performed as previously described 7.

### CFTR activity measurements

CFTR activity was measured in transiently transfected HEK293 cells using the halide‐sensitive yellow fluorescent protein YFP‐H148Q/I152L. Cells were transfected before being transferred to poly‐L‐lysine coated 96‐well black/clear bottom microplates. The next day, cells were washed with PBS and incubated for 30 min with 50 *μ*L of PBS containing 100 *μ*mol/L cpt‐AMPc. Plates were then transferred to a TrisStar plate reader (Berthold, Thoiry, France), and cell fluorescence (excitation: 485 nm; emission: 535 nm) was continuously measured before and after addition of 100 *μ*L of PBS‐NaI (PBS solution where NaCl is replaced with NaI). Signal decay was fitted to an exponential function to derive the maximal slope corresponding to initial influx into the cells. Maximal slopes were converted to rates of change in intracellular I^−^ concentration (in mmol/L/sec).

## Results

### *CFTR* gene analysis and *in silico* analysis

**Figure 1:**
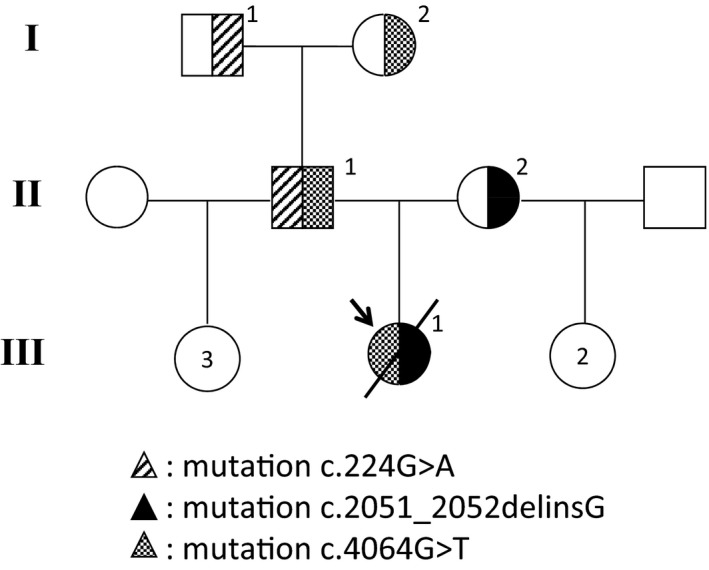
Pedigree and mutations identified in the family.

Extensive *CFTR* gene analysis led to identify the c.4064G>T, p.Cys1355Phe variant in exon 25, in addition to the known CF‐causing variant c.2051_2052delinsG. No other variant or large rearrangement was detected. Analysis of the parents showed that the two variants were *in trans*, p.Cys1355Phe being inherited from the father (Fig. 1). The father was also found to carry c.224G>A, p.Arg75Gln (R75Q) on the other chromosome.

The p.Cys1355Phe variant was not found in *CFTR* mutation databases, CFMDB, and CFTR‐France Database 8. Additionally, it was not reported in dbSNP or in 1000 Genomes Project. It was found only once in a non‐Finnish European population (1/66732 alleles, 0.0015%) in the Exome Aggregation Consortium. *In silico* analysis showed contrasted results: the variant was predicted either deleterious (Grantham, SIFT, PolyPhen2, and Mutation Taster) or neutral (GVGD, KD4v). The amino acid was well conserved (through eleven species) as well as the nucleotide. No effect on splicing was predicted.

**Figure 2:**
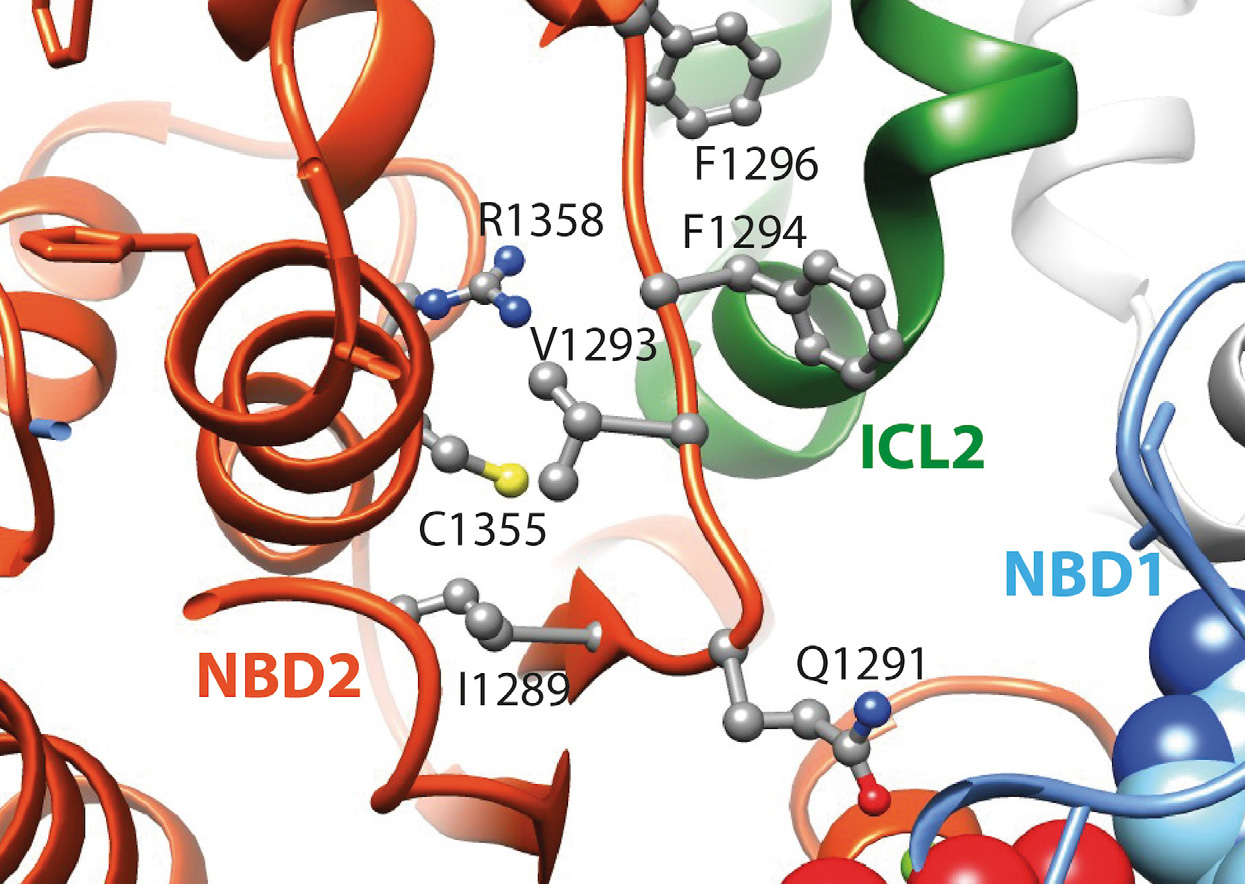
Model of the CFTR‐WT membrane‐spanning domain (MSD) and nucleotide‐binding domain 2 (NBD2) 3D structure.

In the 3D model, residue Cys1355 is buried within the NBD2 core, facing the Q‐loop and in contact with Ile1289 and Val1293 (Fig. 2). The p.Cys1355Phe mutation is predicted to slightly destabilize the NBD2 fold, due to its bulkier side chain. This could have a significant impact on the conformation of the Q‐loop of NBD2 and the function of amino acid Gln1391, which is located within the canonical ATP‐binding site. Additionally, the p.Cys1355Phe mutation could also induce positional alterations of amino acids Phe1294 and Phe1296, residues involved in the aromatic NBD2 groove in contact with intracytoplasmic loop 2 (ICL2).

### Evaluation of c.4064G>T, p.Cys1355Phe on splicing

**Figure 3:**
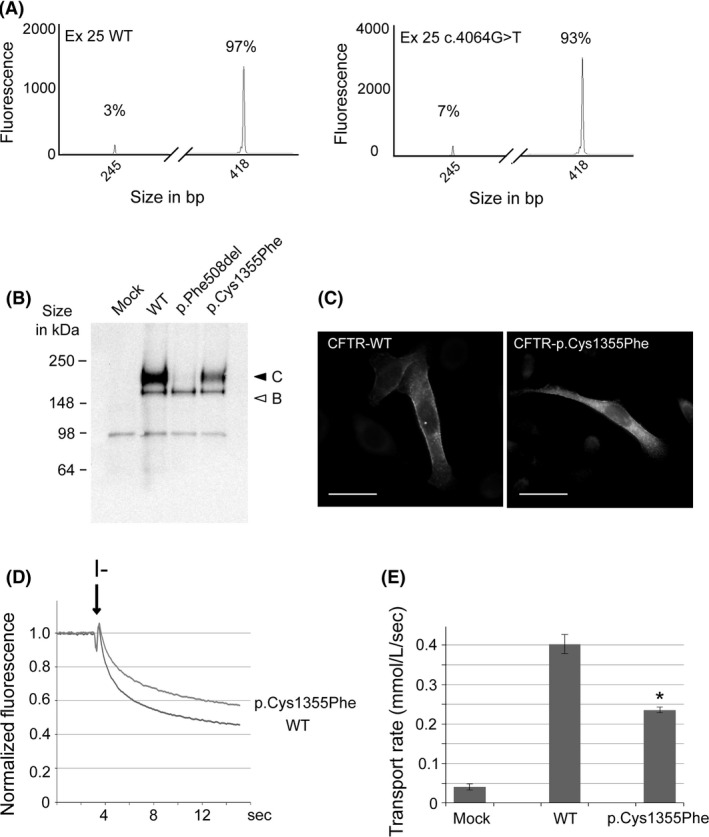
(A) Analysis in fluorescent capillary electrophoresis of RT‐PCR products after minigene experiments obtained after 22 cycles. RNA was purified from BEAS‐2B cells transfected with a minigene containing WT exon 25 or carrying the c.4064G>T substitution. The corresponding size and relative amount of each peak are indicated. (B) Western blot analysis of HeLa cells transiently transfected with the indicated CFTR construct. Core‐ and fully glycosylated CFTR (band B and C, respectively) are indicated by arrows. (C) Immunocytochemistry performed on transiently transfected HeLa cells with the indicated CFTR construct. Scale bar represents 10 *μ*m. (D) Representative cell fluorescence recordings from HEK293 cells transiently expressing the indicated CFTR construct and the halide‐sensitive yellow fluorescent protein (YFP). Addition of PBS‐NaI (I^−^, arrow) caused YFP quenching with a rate proportional to the rate of I‐ influx and CFTR activity. (E) Summary of data obtained from the functional assay reporting rates of I^−^ transport for the indicated constructs and treatment in HEK293 cells. The bars report the transport of iodide as determined from the maximal rate of cell fluorescence decrease (mean ± SE,* n* = 5). The * indicates statistical significance (*p*<0.01).

Exonic point sequence variations can induce exon skipping by altering the binding of SR‐proteins or hnRNPs. These binding sites are not well characterized and are therefore still difficult to predict using *in silico* tools. To evaluate an effect on exon recognition, minigenes containing either WT exon 25 or harboring the c.4064C>T variant plus approximately 300 nucleotides of the flanking intronic region were constructed. After transfection in BEAS‐2B cells and total RNA purification, samples were amplified by RT‐PCR and analyzed by capillary electrophoresis. From this expression system, a single 245‐bp transcript was amplified from the empty plasmid, as previously reported 7. In constructs containing WT exon 25, two peaks could be detected: a major peak at 418 bp corresponding to the full‐length mRNA (97% ± 0.7, *n* = 5) and a minor peak corresponding to exon 25 skipping (3% ± 0.7, *n* = 5). In regard, exon skipping was slightly but significantly increased up to 7% ± 0.3 (*n* = 5, *P* < 0.01) when using the construct containing the c.4064G>T variant (Fig. 3A). Exon 25 skipping would lead to a frameshift and occurrence of a stop codon at the sixth codon of exon 26, leading to the loss of 154 amino acids.

### Evaluation of p.Cys1355Phe on protein maturation and function

The maturation of the CFTR channel is a complex multistep process. WB analysis of the CFTR‐WT protein enables to monitor the glycosylation of the protein and the visualization of both core‐ and fully glycosylated CFTR (B and C band, respectively). HeLa cells were transiently transfected, and maturation efficiency was measured using the C/(B+C) ratio which was of 0.83 ± 0.11 (*n* = 5) for CFTR‐WT and 0.17 ± 0.07 (*n* = 5) for CFTR‐p.Phe508del (Fig. 3B). Point sequence variants can induce maturation defects of the protein and reduction of the fully glycosylated mature CFTR, as CFTR‐p.Phe508del does (Fig. 3B). The maturation efficiency of CFTR‐p.Cys1355Phe was significantly reduced to 0.64 ± 0.13 (*n* = 5, *P* < 0.05), as compared to CFTR‐WT, but partially conserved in comparison with CFTR‐p.Phe508del (*P* < 0.01) (Fig. 3B).

Immunohistochemistry was performed on transiently transfected HeLa cells to monitor CFTR subcellular localization. Cells expressing either CFTR‐WT or CFTR‐p.Cys1355Phe presented similar patterns, although a more marked intracellular staining for CFTR‐p.Cys1355Phe (Fig. 3C), in concordance with biochemical data.

We also performed a YFP‐based functional assay to measure CFTR function in transiently transfected HEK293 cells. Upon addition of a PBS‐NaI solution, YFP fluorescence was quenched by the flow of I^−^ within the cell through active CFTR channels (Fig. 3D). Quenching was not observed in cells transfected with empty plasmid (data not shown). When comparing to CFTR‐WT‐transfected cells, CFTR‐p.Cys1355Phe transfected cells presented reduced fluorescence quenching. Calculated transport rates indicated that CFTR‐p.Cys1355Phe had an activity reduced by half (0.23 ± 0.01 mmol/L/sec, *n* = 5, *P* < 0.01) compared to CFTR‐WT (0.40 ± 0.02 mmol/L/sec, *n* = 5) expressing cells (Fig. 3E).

## Discussion

Establishing the clinical significance of sequence variants may be challenging in a number of situations, especially when it impacts on genetic counseling and reproductive options for a couple. Particularly in CF and CFTR‐RD, varied functional consequences of variants are observed, from no to a severe effect, and true CF‐causing variants are found in all phenotypes. In our case, the fatal and rapid outcome of the premature newborn prevented sweat testing and other electrophysiological, ex vivo testing. By contrast, the father, who had symptoms suggestive for a CFTR‐RD, was found to be compound heterozygous for p.Cys1355Phe and p.Arg75Gln, a known frequent variant not considered as CF‐causing but which was recently shown to impair bicarbonate permeation and increase the risk for pancreatitis 9. This observation supports the potential clinical impact of p.Cys1355Phe. The use of bioinformatics tools to evaluate its impact was poorly contributive, with contrasted possible effects on the protein. From the 3D prediction however, it was tempting to speculate that a maturation defect could implicate both a less stable NBD2 fold as well as an altered interaction between ICL2 and NBD2. The variety of functional tests used eventually led to show cumulated effects: a slight effect on RNA splicing, which is not considered *per se* as clinically relevant, and a reduction in ion transport activity (50% of that of the wild type, *P* < 0.01) that could be explained by a significant decrease in the amount of the fully glycosylated functional channel (64% of that of the wild type, *P* < 0.05). Overall, given the level of residual activity, it is likely that these effects, even in combination, are not sufficient to consider the variant as CF‐causing, but possibly as a CFTR‐RD‐associated variant.

It is therefore difficult to assign the particularly severe clinical manifestations in the infant to the *CFTR* genotype. Great prematurity may be the main cause. Moreover, such severe manifestations have not been documented in premature CF newborns. This study is of importance for genetic counseling matters, for the parents as well as for the father's children and other relatives, who may nevertheless be reassured on their risk of having a CF child.

This study clearly illustrates the contribution of functional studies in the diagnostic process, although they are not part of a routine process in diagnostics laboratories, especially when no in vivo or ex vivo test can be performed. It further illustrates the fact that a single variant can induce multiple defects, altering mRNA synthesis, protein maturation or function. Therefore, evaluation of novel variants needs to combine a variety of in vitro assays. Eventually, this challenges the functional classification of mutations 10, for which there has been renewed interest because of pharmacological therapy.

## URLs

Cystic Fibrosis Mutation Database (CFMDB), http://www.genet.sickkids.on.ca/cftr/

*CFTR* **‐**France Database, https://cftr.iurc.montp.inserm.fr/CFTR/index.html

NCBI dbSNP build 142 (Oct. 2014) through Ensembl e80, http://www.ncbi.nlm.nih.gov/projects/SNP/

1000 Genomes Project, http://www.1000genomes.org/

Exome Aggregation Consortium (ExAC) database (accessed November 2014), http://exac.broadinstitute.org/

## Conflict of Interest

All authors declare the absence of conflict.

## Authorship

AH: involved in conception and realization of the experiences, analysis of the generated data, and writing the manuscript. MPR: involved in the supervision of genetic analysis, coordination of the project, and writing the manuscript. IC: involved in analysis of the CFTR‐3D structure and writing the manuscript. CZ: involved in genetic counseling of the family and drawing the pedigree. BC: involved in supervision and analysis of experiments. JG: is pediatrician of the neonate and involved in writing the manuscript. AI: involved in supervision of the genetic analysis. DL: is department head and received the family for genetic consultation. NM: involved in realization of the experiments. BA: is head of the team and involved in supervision of the project. PF: is head of the team and involved in supervision of the project. PF: involved in supervision of genetic analysis and writing the manuscript. EG: is head of the project and involved in coordination of the project and writing the manuscript.
